# The Design and Implementation of the ECOVIR Project: A Primary Health Care Surveillance System to Strengthen Co-Detection of Respiratory Viruses in Normandy

**DOI:** 10.3390/mps5060098

**Published:** 2022-12-13

**Authors:** Hortense Petat, Matthieu Schuers, Sandrine Corbet, Xavier Humbert, François Le Bas, Christophe Marguet, Lucille Pellerin, Andry Rabiaza, Astrid Vabret, Meriadeg Ar Gouilh

**Affiliations:** 1 INSERM, UMR1311-DYNAMICURE—Institut de Biologie Clinique, CHU, F-7600 Rouen, France; 2 Laboratoire de Virologie, Centre Hospitalo-Universitaire, F-14033 Caen, France; 3 Centre Hospitalier Universitaire de Rouen, Département de Pédiatrie Médicale, EA2656 Université de Normandie, UNIRouen, F-7600 Rouen, France; 4 Département de Médecine Générale, Normandie Université, UFR Santé Rouen, F-7600 Rouen, France; 5 INSERM, U1142, Laboratoire d’Informatique Médicale et d’Ingénierie des Connaissances en e-Santé, LIMICS, Sorbonne Université, F-75006 Paris, France; 6 Département de Médecine Générale, Normandie University, UNICAEN, UFR Santé, F-14000 Caen, France

**Keywords:** primary care /hospital coordination, acute respiratory infections, biobank

## Abstract

Acute respiratory infections (ARIs) need to be better understood and treated, as they are critical to public health, especially during crises such as the SARS-CoV2 pandemic. These are the most abundant infections in the general population and are seen primarily in primary care by general practitioners (GPs). Many different viruses are involved, according to epidemic variations. Viral co-detections account for a significant proportion of ARIs in hospital cohorts. The objective of the ECOVIR cohort was to study viral co-detections by setting up a biobank of respiratory tract samples from patients consulting their general practitioner for ARI symptoms. We report here on the course of the study: the design, the conduct, and the difficulties encountered. ECOVIR (Etude des CO-detections VIrales dans les prélèvements Respiratoires) was a prospective, multicenter cohort conducted in France during two epidemic seasons (2018–2019 and 2019–2020). We recruited GPs. Each GP investigator (GPI) saw patients weekly for examination, clinical data collection, and nasopharyngeal swabbing. Each sample was sent to the virology unit for biobanking and molecular analysis. Clinical and sociodemographic data were collected 7 days after inclusion. ECOVIR involved 36 GPIs. Patients with symptoms of an ARI were included (*n* = 685). The median number of inclusions was 16 patients per GPI over both seasons (IC_25–75%_ [4.75; 27]). Patients aged 18 to 64 years were the most numerous (57%), followed by children (30%), and the elderly (13% over 65 years). This age distribution emphasizes the young adult and middle-aged population. Residents participated in the project and called patients on day 7 to obtain clinical and sociodemographic data. Our study triggered the creation of an original network, which plans to establish a functional link between research and primary health care. Primary care is unfortunately poorly represented in research protocols, particularly in respiratory infections, even though it is a cornerstone of our French health care system, as demonstrated every day in this period of crisis.

## 1. Introduction

Acute respiratory infections (ARIs) are the most common infections in the general population and their rate has been estimated to be between zero and six events per person per epidemic season [1,2]. The annual prevalence of ARIs in France has been approximately quantified at 25 million cases of nasopharyngitis and 10 million cases of bronchitis according to the French Public Health Association. They are mainly caused by viruses [2], but can also be caused by bacteria such as other bacteria and Mycoplasma species [3]. ARIs are mostly community-acquired infections and are managed by general practitioners (GPs). The social and economic costs and morbidity caused by these diseases are considerable: absenteeism, inappropriate and abusive administration of antibiotics, hospitalizations of high-risk populations, and deaths [3]. These detrimental factors have also been highlighted during the current SARS-CoV2 pandemic.

Respiratory viral infections are the primary trigger for exacerbation of chronic respiratory diseases in adults or children, such as COPD (chronic obstruction pulmonary disease) [4,5], asthma, or other diseases [6,7]. They are also at the origin of hospital transmissions in places of collective life, i.e., nurseries or retirement homes. Recurrent exacerbation episodes are well known to alter respiratory trajectories from childhood and to generate a high rate of hospital admissions. However, studies on the etiologies and short-term course of these common respiratory infections in a real-world setting are very limited, and almost all of them have been conducted in emergency departments or during hospital stays.

We therefore conducted a study of viral co-detections in respiratory samples in general practitioners’ offices (project ECOVIR for Etude des CO-detections VIrales dans les prélèvements Respiratoires). Necessary clinical data were collected and nasopharyngeal swabbing was performed to detect any concomitant viral infection. Samples were stored in a virus biobank. Because the implementation of such primary care studies during epidemic seasons is rarely reported, we proposed to describe the successive steps of design, implementation, necessary adaptation to general practitioners’ practices, and difficulties encountered. The clinical and virological data will be the support of other articles.

## 2. Methods and Design

### 2.1. Study Design

#### 2.1.1. Project Team and Infrastructure

**Figure 1:**
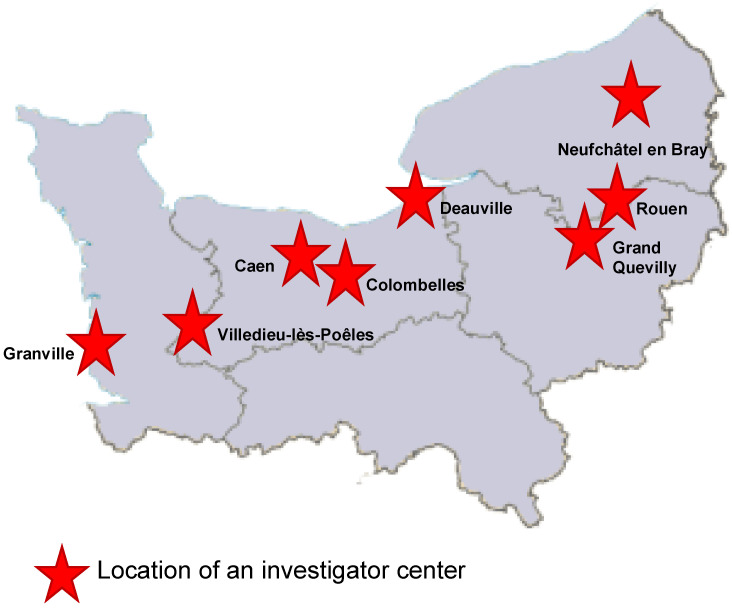
Repartition of the eight investigator centers in Normandy, France.

The ECOVIR project began in May 2018 during the first meeting between GRAM 2.0 (Groupe de Recherche sur l’Adaptation Microbienne) and the heads of the DUMGs (Départements Universitaires de Médecine Générale) of the Universities of Rouen and Caen in Normandy, France. Eleven meetings were held between May 2018 and the start of testing in January 2019. In September 2018, five general practice residents were recruited to participate in the project as part of their medical thesis. At the same time, we selected eight “investigator centers” from among the care centers throughout Normandy (Figure 1). Each center brought together general practitioners.

Our team visited medical centers in September and October 2018 to meet general practitioners (GPs), to introduce them to the ECOVIR project, and to recruit future ECOVIR general practitioner investigators (GPIs). Sampling materials were provided and brought to each GPI. The kit included flocked swabs (COPAN^®^) with a small flexible shaft, a virologic transport holder, and patient handouts. Every GPI received enough material to test 30 patients.

#### 2.1.2. Study Design

**Figure 2:**
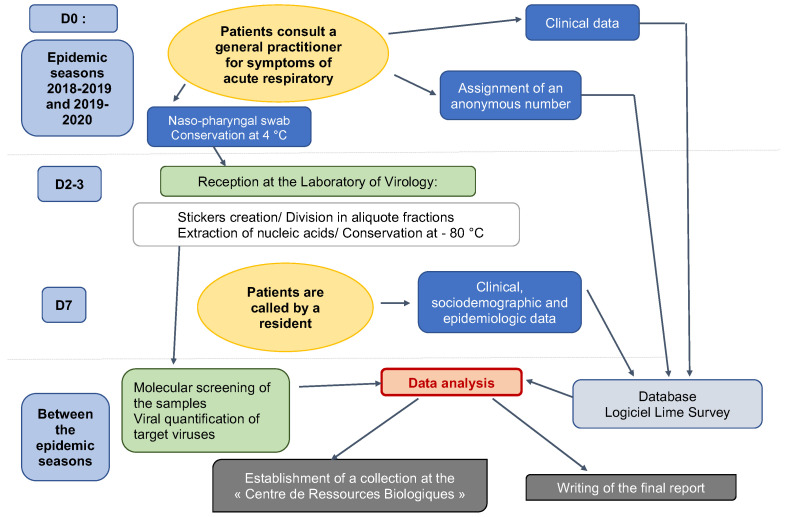
ECOVIR’s workflow.

ECOVIR was a cross-sectional study. Patients were included during a visit to the GP. Inclusion criteria were: French-speaking patients, children or adults, and spontaneously consulting their GP for symptoms of acute upper or lower respiratory infection at the beginning of the week (Monday or Tuesday or Wednesday). The criteria for non-inclusion were: non-French speakers (because of the phone call and data collection on Day 7), people who could not be contacted by telephone, or people with a history of epistaxis. Patients were divided into 6 age groups: 0–23 months old, 2–5 years old, 6–17 years old, 18–64 years old, 65–74 years old, and more than 75 years old. Each GPI was assigned an age class based on their patients’ profile. GPIs were responsible for performing research-related actions (Figure 2): giving information to the patient, taking the patient’s consent to participate in the study, and handing over the information document. The GPIs then collected clinical data, and performed the nasopharyngeal swab and stored it at 4 °C. Samples were collected weekly by our team and brought to the virology laboratory of the Caen hospital, according to a predetermined schedule. This schedule was discussed with the GPIs, according to their absences and unavailability. At the moment of inclusion, patients were informed of a call on day 7 after inclusion, conducted by five residents on our team. Throughout the call, which lasted approximately 5 to 7 min, additional data was collected: personal medical history, environment, and evolution of symptoms. Residents tried to reach patients several times if the first call was unsuccessful. Patients were considered lost to follow-up after nine unsuccessful calls and messages.

**Figure 3:**
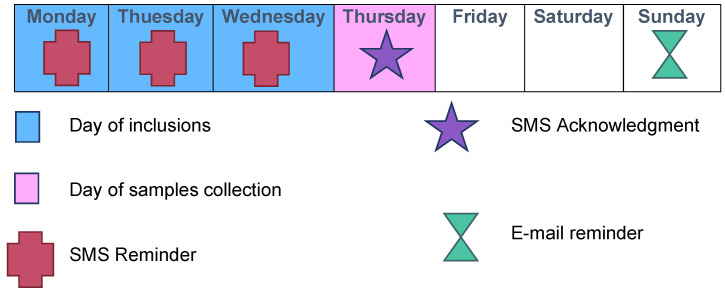
Typical inclusion week during ECOVIR’s project.

Each week was scheduled as follows: inclusions on Mondays, Tuesdays, and Wednesdays; transport of samples to the virology laboratory by our team on Thursdays; and analyses on Fridays (Figure 2 and Figure 3).

### 2.2. Measures and Data Collection

#### 2.2.1. Measure Collection

At inclusion, GPIs briefly collected clinical data via Lime’s Survey^R^ software (https://www.limesurvey.org/, accessed on 1 September 2018). The questions asked about the patients’ clinical status at inclusion, the diagnosis made, and detailed the treatments prescribed. Seven days after inclusion, patients were called by the residents to obtain an accurate medical history of the patients, their environment, and disease progression since the consultation.

Samples were received at the virology unit of the University Hospital of Caen once a week and divided into aliquots. One aliquot was examined by a Multiplex PCR test (NxTag RPP Luminex kit^®^, Austin, TX, USA) after nucleic acid extraction performed on “QIAsymphony” (Qiagen, Hilden, Germany). The viruses searched for were the following: Respiratory Syncytial Virus A and B; Entero/Human Rhinovirus; Adenovirus; Metapneumovirus; Bocavirus; Coronaviruses OC43, NL63, 229E, and HKU1; Influenza A H1 and H3; Influenza B; and Parainfluenza 1,2,3, and 4. Other aliquots were preserved at −80 °C.

#### 2.2.2. Outcome Measure

Clinical data were available for ongoing analysis via Lime Survey^R^ software.

Virological data were reported each week of inclusion and it was not possible for the GPIs to know the virological results.

### 2.3. Intervention

#### 2.3.1. GPI Training for Nasopharyngeal Swab Collection

Each GPI was trained in nasopharyngeal swabbing. The training consisted of a video presentation followed by practice supervised by our experts.

#### 2.3.2. Measures Implemented to Ensure the Feasibility of the Study

For each GPI, the goal was to include 3 patients per week during the inclusion periods. To reduce information time and promote patient adherence, explanatory posters were available in the GPI waiting rooms. The online questionnaire was designed to be completed in approximately 2 to 3 min by the GPI after the physical examination at inclusion.

A daily “pacing schedule” for the GPI was established, with text messages throughout the inclusion period (Figure 3). Each Thursday, an e-mail and a text message were sent to the GPIs to thank them for their participation in the study and to inform them in real-time of the number of completed inclusions. A permanent team was available to answer questions from the GPIs regarding research, technical, or logistical issues.

#### 2.3.3. Assessment at the End of the First Season

To better understand the insufficient number of inclusions or disparities observed in the first epidemic season, we organized meetings with the GPIs during the summer between the two epidemic seasons. We met with each GPI and an anonymous questionnaire was sent to each GPI.

### 2.4. Ethics

After four meetings in the summer of 2018 between the GRAM team and the DUMG, for scenario adjustment, the final protocol was submitted to the “East II” Protection Committee (study reference 18/10/10/63004) on 10 October 2018. The protocol package was forwarded to the commission on 19 November 2018. A favorable opinion was given with minor modifications. A second packet was forwarded to the commission on 12 December 2018, which responded positively on 7 January 2019.

An information document was given to each included patient who participated in the study (a no objection document). The document was adapted according to the age of the included patient. For children under 6 years old, an information document was adapted with drawings and pictograms. It was read and given to children by the GPI.

## 3. Results

### 3.1. GPIs

ECOVIR was conducted over two epidemic seasons: the first from 21 January to 10 April 2019 (12 weeks) and the second from 4 October 2019 to 13 March 2020 (21 weeks). The second season ended earlier due to the lockdown of the SARS-CoV2 pandemic in France. Eight investigating centers participated in the study. Thirty-three IPGs were involved in the first season and 36 in the second.

Each GPI from the first season confirmed their participation to the second season. Residents’ supervisors represented 74% of the GPIs involved. The median age of the GPIs was 44 years and women represented 42% of the workforce. They worked an average of seven half-days per week for 14.6 years.

### 3.2. Patients Included

**Figure 4:**
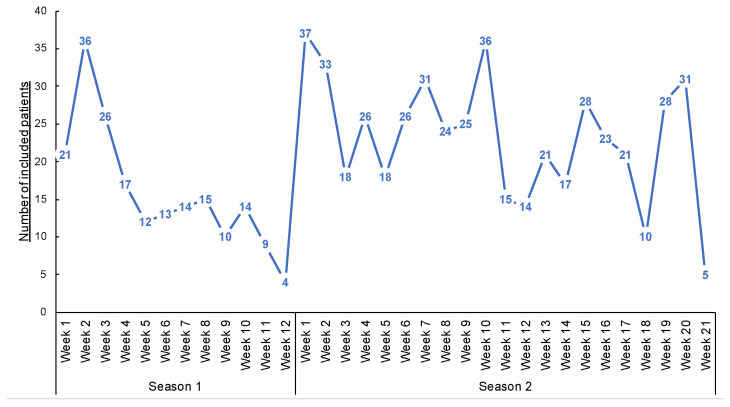
Number of inclusions during the weeks of inclusions.

**Figure 5:**
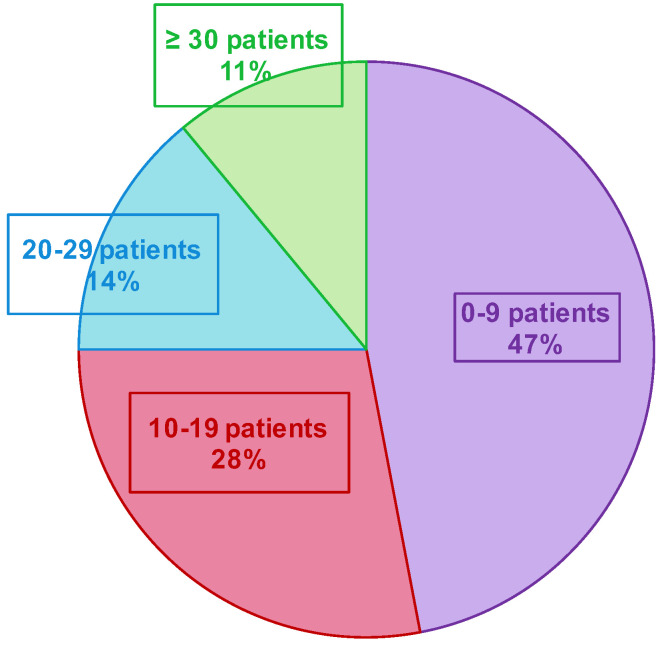
Number of included patients by the GPI during the two epidemic seasons.

A total of 685 patients were included in both seasons (191 and 494 in the first and second seasons, respectively—Figure 4 and Table 1. Three patients declined to participate in the study (e.g., parents cited the potential pain of nasopharyngeal swabbing on their child). Several patients were included multiple times in both seasons. Over the two seasons, the median inclusion was 16 patients per GPI (IC_25–75%_ [4.75; 27]) (Figure 5). During the 12 weeks of inclusion in the first season, 24 GPIs (out of 33 enrolled) included patients (six patients over the 12 weeks on average). During the 21 weeks of the second season, 31 (of 36) GPIs included patients. A total of 10 GPIs required additional specimen collection kits, while 75% of GPIs attributed the lack of inclusions to oversight. Nevertheless, the frequency of reminders (SMS and e-mails) was judged “adequate” by 100% of the GPIs. IPMs assessed the ease of collection for each patient included: 8.7% of collections were described as “not easy” during their practice.

Patients aged 18–64 years were the most numerous (57%), followed by children (30%) and the elderly (13% were over 65 years). This age distribution emphasizes the young adult and middle-aged population that are not typically seen in hospitals.

### 3.3. Virologic Analysis

A total of 672 samples (98% of inclusions) were collected at the laboratory. We noted some logistical problems in getting the samples to the laboratory, mainly due to unforeseen absences of GPIs. An amount of 627 specimens were described “with ease” by the IPMs who performed them (91%). Of the total samples brought to the laboratory, 447 (67%) were positive for at least one respiratory virus targeted by the Multiplex PCR test.

### 3.4. Phone Call on Day 7

Regarding the call on day 7, 631 (92%) patients responded. We noted an increase in the number of people lost to follow-up during school vacations (Christmas and February of each season), as well as a decrease in inclusions during the same periods. Each patient called had been informed of the call by the GPIs. No patient refused to answer the telephone questionnaire.

### 3.5. Intercurrent Events

We recorded five calls from the GPIs to our team. The first concerned an Internet problem; the second, a question about an inclusion criterion; two mentioned a patient included for the second time; and in the last one, the GPI asked if we could give the patient the virological result on day 7.

## 4. Discussion

Our work is an original study involving the implementation of a coordination between hospital, primary health care, and practitioners of many specialties. Through the DUMG, our team included 685 ambulatory patients distributed all over Normandy and analyzed the nasopharyngeal swabs over two epidemic seasons.

We have created an ambulatory/hospital network around a non-interventional research project. Nevertheless, the swabbing act was sometimes considered potentially invasive and was not easily accepted before the SARS-CoV2 pandemic. Despite this relative acceptance, we noted an excellent inclusion level with only three patients who refused to be included in the study.

The GPIs were younger than the regional mean age of the GPs (44 versus 54 years old), more often female (42% versus 36%), and worked for the majority in part-time work (7 half-days per week) versus 11% in France according to the DREES (Direction de la Recherche, des Etudes, de l’Evaluation et des Statistiques, Direction of Research, Studies, Evaluation and Statistics). Thanks to new communication technologies (SMS and emails), we could exchange continuously and very easily with the GPIs and we could immediately attend to their difficulties or their requests. The median age of the GPIs, mostly belonging to so-called “generation Y”, likely explains the effectiveness of emails and SMS exchanges as well as online questionnaires.

It was difficult to compare our methodology with other cohorts, French or international, because primary health care is not often represented in virological clinic published studies. We focused on the experiences and successes of the GROG network to organize this new research network. Between 1984 and 2014, during 30 winter epidemic seasons, some GPs participated in the “GROG” (Groupes Régionaux d’Observation de la Grippe, Flu Regional Observation Groups), forming a flu-monitoring network in primary health care, a symbol of the GPs’ participation to French Public Health. The GROG were present in 21 out of 22 French regions and followed actively, every winter, the progress of flu epidemics, including patients with ARIs and consulting their GP. The GROG network was an epidemiological monitoring network and was more dedicated toward surveillance than research. Health systems are different in every country and not easily comparable. For example, American cohorts of ambulatory patients with acute respiratory infections [8,9,10] included patients who went to emergency departments. This is a very different recruitment compared to studies carried out in primary health care as defined in ECOVIR or more broadly in France. In 2018, a study focusing on the etiology of lower respiratory infections took place at the European scale, but no methodology describing the implementation of the study was available and the 294 GPs included only about 10 patients each over a period of 3 years [11].

The implementation of such a research program and network is not usual. The concertation with GPs, the practical feasibility of the study, and the necessary adaptation to GPs’ activity are key factors to anticipate and discuss. In our ECOVIR project, the initial objective was to include three patients per week and per GPI, but only 69% of the expected number of included patients was effectively included. We also observed an unforeseen heterogeneity of inclusion rate among the 84% of GPIs that included patients (vs. 73% for the GROG in 2014). A strong heterogeneity in the numbers of inclusions among GPs in the GROG network was found as well. During the last year (2014), they described 1/3 of “small samplers”, 1/3 of “intermediate samplers”, and 1/3 of “big samplers”. In our study, we rather observed 75% of “small samplers”, 14% of “intermediate samplers”, and 11% of “big samplers”. In the GROG study, the GPIs had to include the first eligible patient of the week, every week. We first planned to do the same, but the DUMG preferred not to impose the inclusion schedule to the GPs and to choose the right moment in the day to make a “better rate inclusion”. The lack of inclusions by the GPIs cannot be explained by the lack of eligible patients because according to the OMG (Observatoire de Médecine Générale, General Medicine Observatory), the reasons, “febrile condition” and “common cold” (that stand for main ECOVIR’s inclusion criteria), represent 17 and 12%, respectively, of the reasons to consult the general practitioners. A proportion of 75% of the GPIs confessed to have forgotten the study, whereas there was an eligible patient at their consultation. The loss of time is a frequent reason not to include patients during a consultation. The median duration of a consultation in general medicine in France is 17 min. We estimated that about 6 min is the necessary time to include a patient in ECOVIR. This had an impact in the progress of a journey. Moreover, we noted that the general practitioners that participated in the GROG could know the virologic results of the patient they had sampled, unlike our GPIs who had no personalized result, ever. This reflects an important willingness from the GPIs to participate in clinical and virological research.

Involvement of young researchers, including PhD students, residents of general medicine, and the multidisciplinary team, were key to the development and the implementation of the project.

We noted a significant reluctance to the practice of nasopharyngeal swab collection among the GPs. This was a limit to the inclusions, particularly for young children. Nevertheless, only 8.7% of the samples were described as “not easy” during their practice. Training in the nasopharyngeal swabbing procedure allowed GPIs to include and collect samples in a comfortable manner. We found that the more patients GPIs included, the easier they found the nasopharyngeal swabbing procedure. This virtuous circle did not exist for all GPIs because some of them remained on their first apprehension of the procedure. This apprehension was still present during the initial swabs for all GPIs, but most GPIs eventually concluded that performing the swab was simple and quick. It is interesting to note that in France and before the pandemic, this nasopharyngeal swab procedure was only performed in hospitals and never in primary care.

The relatively low number of inclusions could also be explained by the limitation of inclusions to 3 days per week due to the IPG schedule and sample storage issues. Indeed, a significant portion of GPIs worked part-time, with approximately 50% of IPMs not working on Wednesdays. Other disruptive events should also be noted, such as vacations, time off for training, and periods of overactivity. The SMS reminder mode was deemed “effective” and “adequate” by the GPIs, but other more interactive modes of communication, promoting a bottom-up approach with active IPG input, would be very beneficial. Social networking tools could be an interesting alternative to SMS by allowing any active participant to interact with the group.

We tried to anticipate the difficulties and put measures in place to facilitate the feasibility of the study. However, the analysis of this first implementation highlighted the need to refine the feasibility criteria according to the GPs’ mode of practice, in order to specify the evaluation of the study’s limits.

Our study is the first in France to look at the frequency of viruses in primary care patients: 67% of samples were positive for one or more viruses in the Multiplex PCR test for the viruses studied. However, this rate of viral detection is known in the hospital through the analysis of nasopharyngeal swabs. In addition, most studies have focused on patients with conditions requiring hospitalization. As in the hospital cohorts, the rate of negative samples is significantly higher in adults (40% in the 18–64 age group) than in children (8% in the 2–5 age group) (EPIC study) [8,9]. However, in this multicenter study, the control group consisted of patients without symptoms, raising the question of asymptomatic viral infections. In our study, there was no asymptomatic patient because of the inclusion criteria. In the Tennessee Children’s Respiratory Initiative (TRCI) study [10], 26% of the samples, which came from patients with high ARIs, were negative, but the cohort was limited to infants less than 12 months old.

The strength of this project was to demonstrate the feasibility of a study with samples from the upper airway, in a large cohort, with less than 8% of patients lost to follow-up at day 7. This study allowed us to obtain an initial cohort of patients in primary care. The creation of this network allowed us to communicate during the pandemic, which disrupted our study. We hoped to continue inclusions, but at the early stage of the pandemic, we felt that the GPIs could not safely perform the nasopharyngeal swab, although they requested it. In the future, we will know how to better communicate with primary care, sending regular reminders. We think that nasopharyngeal swabbing has become a daily care since the beginning of the pandemic.

## 5. Conclusions

Our project, ECOVIR, is unique and included 685 patients of all ages with acute respiratory infection in primary care. This has created a strong collaboration between the hospital, 36 investigating general practitioners, spread throughout Normandy, France, and our research team (GRAM 2.0). It has created a new network to link research to primary care, which is a central part of the French health system and is poorly represented in research protocols.

## Figures and Tables

**Table 1:** Characteristics of the patients.

		n (%)
Age groups	0–24 months 2–5 years 6–17 years 18–64 years 65–74 years >75 years	76 (11) 58 (8) 72 (11) 388 (57) 61 (9) 30 (4)
Female sex		405 (59)
Patients lost to follow-up		53 (8)
Analyzed samples		672 (98)
Samples positives in Multiplex PCR test		447 (67)

## Data Availability

Data is kept by the “Direction de la Recherche” of the Caen’s CHU, France.

## Study Status

The final protocol was submitted to the “East II” Protection Committee (study reference 18/10/10/63004) on 10 October 2018. The protocol package was transmitted to the committee on 19 November 2018. A favorable opinion was given with a request for minor modifications. A second submission was sent to the commission on 12 December 2018, which responded positively on 7 January 2019. The first inclusion took place on 21 January 2019.

## Abbreviations

ARI	acute respiratory infections
COPD	chronic obstructive pulmonary disease
ECOVIR	Etude des CO-détections VIrales dans les prélèvements Respiratoires—Study of viral co-detections in respiratory samples)
DUMG	Départements Universitaires de Médecine Générale, General Medicine University Departments)
DREES	Direction de la Recherche, des Etudes, de l’Evaluation et des Statistiques, Direction of Research, Studies, Evaluation and Statistics
GP	general practitioner
GPI	general practitioner investigator
GRAM	Groupe de Recherche sur l’Adaptation Microbienne, Microbial Adaptation Research Group
GROG	Groupes Régionaux d’Observation de la Grippe, Flu Regional Observation Groups
OMG	Observatoire de Médecine Générale, General Medicine Observatory
PCR	polymerase chain reaction
